# Evolution and Current Status of Influenza A Virus in Chile: A Review

**DOI:** 10.3390/pathogens12101252

**Published:** 2023-10-17

**Authors:** Marcos Godoy, Marco Montes de Oca, Diego Caro, Juan Pablo Pontigo, Molly Kibenge, Frederick Kibenge

**Affiliations:** 1 Centro de Investigaciones Biológicas Aplicadas (CIBA), Puerto Montt 5480000, Chile; marco.montesdeoca@ciba.cl (M.M.d.O.); diego.caro@ciba.cl (D.C.); 2 Laboratorio de Biotecnología Aplicada, Facultad de Ciencias de la Naturaleza, Escuela de Medicina Veterinaria, Sede de la Patagonia, Universidad San Sebastián, Puerto Montt 5480000, Chile; juan.pontigo@uss.cl; 3 Department of Pathology and Microbiology, Atlantic Veterinary College, University of Prince Edward Island, 550 University Ave, Charlottetown, PE C1A 4P3, Canada; mkibenge@upei.ca

**Keywords:** influenza A virus, avian influenza virus, genomic surveillance, poultry farms, wild birds, HPAI H5N1

## Abstract

The influenza A virus (IAV) poses a significant global threat to public health and food security. Particularly concerning is the avian influenza virus (AIV) subtype H5N1, which has spread from Europe to North and Central/South America. This review presents recent developments in IAV evolution in birds, mammals, and humans in Chile. Chile’s encounter with IAV began in 2002, with the highly pathogenic avian influenza (HPAI) H7N3 virus, derived from a unique South American low pathogenic avian influenza (LPAI) virus. In 2016–2017, LPAI H7N6 caused outbreaks in turkey, linked to wild birds in Chile and Bolivia. The pandemic influenza A (H1N1) 2009 (H1N1pdm09) virus in 2009 decreased egg production in turkeys. Since 2012, diverse IAV subtypes have emerged in backyard poultry and pigs. Reassortant AIVs, incorporating genes from both North and South American isolates, have been found in wild birds since 2007. Notably, from December 2022, HPAI H5N1 was detected in wild birds, sea lions, and a human, along Chile’s north coast. It was introduced through Atlantic migratory flyways from North America. These findings emphasize the need for enhanced biosecurity on poultry farms and ongoing genomic surveillance to understand and manage AIVs in both wild and domestic bird populations in Chile.

## 1. Introduction

Influenza viruses belong to the Orthomyxoviridae family [1], and, along with the Amnoonviridae family [2], they constitute the order Articulavirales [3]. Members of this family possess a segmented negative-sense single-stranded RNA genome. The number of genome segments varies, depending on the genus: Orthomyxoviridae has 6–8 segments, while Amnoonviridae has 10. Influenza viruses are classified into four genera: Alphainfluenzavirus, Betainfluenzavirus, Gammainfluenzavirus, and Deltainfluenzavirus. Each genus has a single ratified species: influenza A virus (IAV), influenza B virus (IBV), influenza C virus (ICV), and influenza D virus (IDV), respectively [4]. The Orthomyxoviridae family includes not only influenza viruses but also other orthomyxoviruses categorized into different genera, such as Thogotovirus, Isavirus, Mykissvirus, Sardinovirus, and Quarajavirus [1,4,5,6]. Most recently, the reclassification of the genus Quarajavirus as the family Quaranjaviridae has been proposed. Additionally, a novel and divergent family, tentatively named Cnidenomoviridae, has been discovered in Cnidaria (including corals) and is now included in the order Articulavirales, bringing the total number of families in this order to four [7].

IAV is the most variable genetically and with the widest natural host range of the four species of influenza viruses [8]. IAVs are divided into subtypes based on the antigenicity of their two envelope glycoproteins: haemagglutinin (HA) and neuraminidase (NA) [9]. To date, 16 HA subtypes (H1–H16) and 9 NA subtypes (N1–N9) have been described [8,9], and all combinations, a total of 144 IAVs subtypes, can occur in birds; the migratory wild aquatic birds are considered the primordial reservoir of all influenza viruses for avian and mammalian hosts [9,10,11]. Two new influenza A-like virus subtypes, designated “H17N10” and “H18N11”, have been detected only in bats from Central and South America [12,13]. Additionally, the recent detection of transcripts of a highly divergent influenza virus in a Siberian sturgeon (*Acipenser baerii*) suggests fish are among the first hosts of influenza viruses [7].

Influenza viruses infect many wild birds and mammals, including humans, adapting to new hosts and forming several lineages specific to humans, horses, swine, dogs, and other animals [14]. Thus, depending on their origin host, IAVs can be classified as avian influenza viruses (AIVs), swine influenza viruses (SIVs), or other animal influenza viruses. In human populations, IAVs and IBVs cause seasonal epidemics (known as flu season), whereas IAVs can cause pandemics when gene reassortment mutations result in the emergence of new IAVs against which people have little or no immunity [15]. In bird populations, AIVs that cause mild disease in poultry are referred to as low pathogenic avian influenza (LPAI) viruses. These viruses can mutate into the highly pathogenic avian influenza (HPAI) phenotype, which causes severe disease and high mortality in poultry. The mutations leading to HPAI frequently occur in the AIV H5 and H7 subtypes [16]. Since 2022, outbreaks of HPAI H5N1 viruses have been detected first in Europe and then in North America [17] and subsequently in South America in commercial poultry, backyard poultry, wild birds, and mammals, including humans [18,19]. This is an unprecedented occurrence. The current HPAI epidemic persists, with 38 poultry-related outbreaks and 152 incidents involving non-poultry avian and mammalian species reported globally, except in Oceania, according to the World Animal Health Information System situation report covering the period from 14 July to 24 August 2023 [20]. Approximately 230,000 poultry birds perished or were culled during this time. Outbreaks are anticipated to decline, particularly among poultry. However, sporadic non-poultry avian incidents continue, like the recent sizable mortality event in Norway’s Troms Og Finnmark region, where 12,000 black-legged kittiwake birds succumbed to the virus. The ongoing circulation of the virus in all global regions, except Oceania, remains a significant concern, encompassing both poultry and other animal species [20]. This review provides a summary of the latest developments in the evolution of different IAV subtypes in birds and mammals, including humans, in Chile, with an emphasis on the current status of HPAI H5N1 viruses (Supplementary Table S1).

## 2. Influenza A Virus Subtypes in Commercial Poultry

### 2.1. AIV H7N3

**Figure 1:**
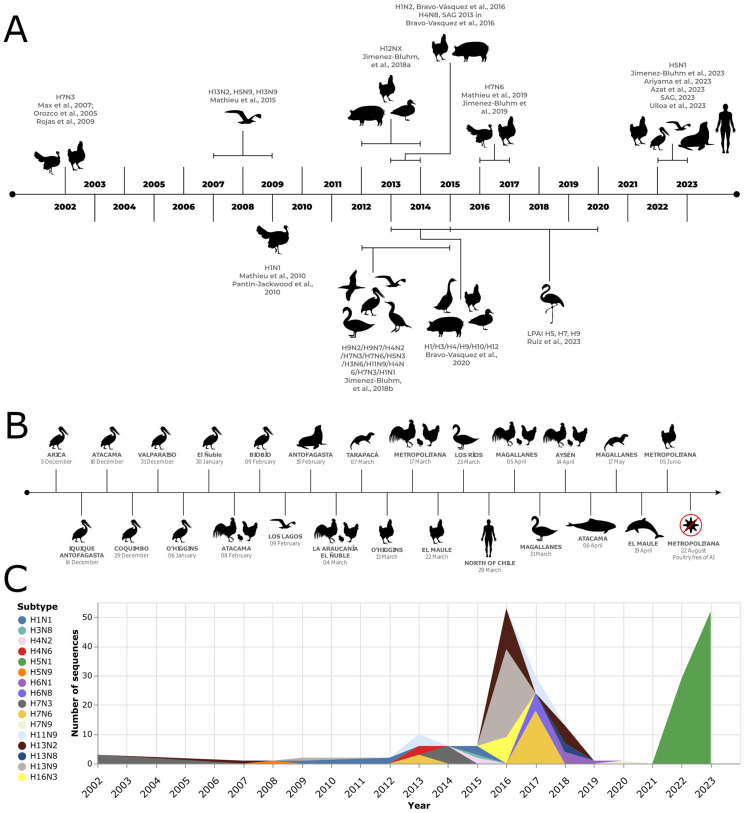
Influenza A virus outbreak in birds and non-human mammals in Chile. (**A**) Literature review derives a timeline of outbreaks [21,22,24,25,26,27,28,29,30,31,32,33,34,35,36,37,38,39]. (**B**) H5N1 cases were registered during late 2022 and 2023, based on data from the Chilean Agricultural and Livestock Service [36] and the National Fisheries and Aquaculture Service [40]. (**C**) Number of hemagglutinin sequences available from avian samples and classified by subtype per year in Chile. Only the three most abundant subtypes per year are shown. Sequences retrieved from the NCBI nucleotide database [41].

The first IAV disease occurrence in commercial poultry in Chile was in 2002 and was caused by HPAI H7N3 (Figure 1), characterized as lineage A/chicken/Chile/184240-4322/2002(H7N3), which had mutated from an LPAI virus [21]. The epicenter of the outbreak was a broiler breeder farm in the densely populated poultry region of San Antonio, V Region, Chile. The farm consisted of 27 poultry sheds housing birds ranging from 1 to 79 weeks in age, besides a hatchery [22]. Between April and May 2002, the farm experienced a clinical disease characterized by a relatively low mortality rate, a slight decline in egg production, and cases of salpingo-peritonitis. In certain cases, mortality was so sudden that no clinical signs were observable. The necropsy revealed cyanotic combs and wattles, as well as petechial hemorrhages in various organs, such as the muscles, heart, pancreas, and legs. Subcutaneous edema was also present. Subsequent surveillance activities identified a second outbreak occurring one week later at a turkey breeding farm owned by the same company. This farm, situated 4 km from the initial outbreak, consisted of eight pens that housed turkeys ranging in age from 6 to 59 weeks, besides a hatchery. The infection was confined to 25% of the sheds. Clinical signs predominantly affected the upper respiratory tract and were followed by a sudden increase in mortality. Cloacal swabs were collected, and an HPAI virus was isolated from two samples from this farm, which were identical to those obtained from the index case [22].

The initial identification of LPAI H7N3 was followed by the detection of HPAI H7N3, which was accompanied by increased mortality rates. Detailed analysis of the viral genomes has yielded valuable insights, revealing minimal genetic differences between the low and highly pathogenic strains, except for a significant alteration in the cleavage site of the HA protein. The LPAI H7N3 virus exhibited a cleavage site similar to that of other LPAI H7 viruses. In contrast, the HPAI H7N3 isolates displayed a 30-nucleotide insertion at this specific site. This insertion possibly resulted from recombination events between the HA and nucleoprotein genes of the LPAI H7N3, leading to an increase in pathogenicity. A comparative analysis of the full sequences of the eight gene segments confirmed that the Chilean viruses had a distinct nature vis-a-vis other AIVs, forming a unique clade exclusive to South America. These findings highlight the critical importance of the continuous monitoring and surveillance of AIVs. These viruses can undergo mutations that significantly increase their pathogenicity [16]. In addition, identifying a distinct viral clade specific to South America underscores the necessity for an understanding of and control strategies tailored to effectively managing AIV outbreaks in the region [23].

Interestingly, isolates obtained from the HPAI H7N3 outbreak in commercial poultry in Chile in May 2002 [22] shared similarity in some genes with an LPAI H7N3 virus isolated from a Cinnamon Teal (*Anas cyanoptera*) specimen in Bolivia in 2001, whose NA and matrix (M) genes shared the highest sequence similarity with North American AIV isolates. The identification of genetic similarities between AIV isolates from wild birds in North America and outbreaks in commercial poultry in Chile further emphasize the importance of the ongoing surveillance and understanding of AIVs in both wild and domestic bird populations [42].

### 2.2. Pandemic Influenza A (H1N1) 2009 (H1N1pdm09)

The pandemic influenza A (H1N1) 2009 (H1N1pdm09) virus, which caused the fourth influenza pandemic [43], emerged as a triple reassortant virus in pigs [44]. In animals, it was first recognized in Canada in May 2009, where it caused respiratory disease in pigs, and in Chile in June 2009, where it led to a significant decrease in egg production in turkeys [25]. The reverse zoonotic virus transmission to turkeys in 2009 remained somewhat a mystery. The artificial insemination of turkeys by infected staff has been proposed as an unusual but possible pathway, considering that, in modern turkey production, turkey hens are handled once a week for intrauterine insemination in order to produce fertile eggs [25]. During the outbreak in turkeys in Chile, there was a decrease in egg production and shell quality among turkey flocks on two farms (A and B) located in the Valparaiso region of Chile. The suspicion of AIV led to the collection of blood samples from the affected turkeys on 14 August 2009, for serological testing [26]. The agar gel immunodiffusion (AGID) assay detected IAV antibodies in 140 out of 227 turkeys sampled, with a higher proportion of positive cases in Farm A (80%) than in Farm B (32%) [26]. Consequently, the Chilean Agricultural and Livestock Service (SAG) implemented control measures, including quarantine, intensified biosecurity measures, epidemiological investigations, and postmortem examinations [26]. Sampling for the detection of viral RNA was conducted on 16 August 2009, on affected flocks, surrounding premises, and neighboring turkey farms. Two days later, the results showed that the infection was present only in the breeder turkeys from the initially affected flocks of Farms A and B. The real-time reverse transcription–quantitative polymerase chain reaction (RT-qPCR) identified RNA corresponding to the IAV M gene but not the H5 or H7 genes. Viral RNA was detected in some cloacal and tracheal swabs but not in the homogenates of turkey embryo lungs and tracheas. This ruled out vertical transmission. Necropsies revealed specific lesions in some birds, while others were recovering. Fecal samples from wild birds near Farm A tested negative for the influenza virus. Subtyping tests identified the virus subtype as H1N1. On 19 August 2009, SAG authorities coordinated with the Chilean Public Health Institute (ISP) to isolate the virus and sequence its genome. The viral sequences exhibited characteristics almost identical to the novel influenza A (H1N1) pdm09 virus [26], suggesting reverse zoonosis, namely the transmission of the influenza A (H1N1) pdm09 virus from humans to turkeys. The virus was classified as belonging to the A/turkey/Chile/28317-6504-3/2009(H1N1) lineage. Follow-up testing indicated that the virus was eliminated from turkeys within 2–4 weeks. During September and October, SAG implemented an RT-qPCR assay to detect the N1 gene of the influenza A (H1N1) pdm09 virus as the flock’s egg production gradually recovered. The most recent evidence of infection was obtained on 31 August 2009, indicating the successful eradication of the virus from the turkeys [26].

### 2.3. AIV H7N6

In December 2016, a turkey farm in the Valparaiso region of Chile was infected with LPAI H7N6. Two weeks later, another turkey farm 70 km north of the first site was also infected. The animals presented a variety of gross lesions, including bursitis, catarrhal to mucopurulent caseous sinusitis, tracheitis, caseous/purulent airsacculitis, polyserositis, pericarditis/hydropericardium, congestion, pulmonary edema, mucopurulent to caseous pneumonia, localized subcutaneous emphysema, mild splenomegaly, pancreatitis, petechiae, ecchymosis focused on the epicardium and coronary fat, fibrin-purulent pericarditis, pulmonary congestion/mucopurulent to caseous pneumonia, and pleuritis. On 28 January 2017, a backyard poultry farm also tested positive for AIV through the AGID test. However, the PCR test result was negative. Various measures, such as depopulation, zoning, animal movement control, and active surveillance, were implemented to contain the outbreak. The 2016–2017 Chilean LPAI outbreak in turkeys involved a group of viruses belonging to a monophyletic clade. These viruses share the closest common ancestor with two other viruses, A/yellow_billed_pintail/Chile/10/2014(H7N3) and A/yellow_billed_teal/Chile/9/2013(H7N6), collected from wild water birds in the same area. The A/turkey/Chile/2017(H7N6) LPAI virus is part of a native South American lineage. Phylogenetic analysis revealed a close relationship between the A/turkey/Chile/2017(H7N6) LPAI viruses and AIV found in wild aquatic birds in Chile and Bolivia, as well as the A/chicken/Chile 2002 virus, which caused the HPAI H7N3 outbreak in Chile. Based on the HA phylogeny, these South American AIVs formed a distinct cluster [27,28].

## 3. IAV Subtypes in Backyard Poultry and Swine

### 3.1. IAV H12NX

Between 2012 and 2014, a surveillance study by Jimenez-Bluhm et al., 2018 [29] employed a comprehensive analysis of serological and molecular data, which revealed the significant presence and circulation of IAV within backyard populations, providing compelling evidence supporting the exposure of poultry in backyard productive systems (BPS) in central Chile to IAV. Using RT-qPCR, IAV RNA was successfully detected in poultry during three out of four sampling seasons, which further confirmed the existence of the virus. Additionally, seroconversion, a confirmatory diagnosis through serology, was observed in 60% of the sampled counties among farm animals, including poultry and swine. These findings collectively highlight the widespread dissemination of IAV among the animal populations studied in Central Chile. Although attempts to isolate IAVs from positive samples were unsuccessful, a complete H12 HA sequence was obtained from a cloacal swab of a domestic Muscovy duck (*Cairina moschata*), and the virus was classified as lineage A/Muscovy duck/Chile/3/2013(H12). The closest sequence comparison revealed 94% identity with an H12 virus obtained from Alberta-Canada in 2003 (A/pintail/Alberta/49/2003) [29].

### 3.2. SIV H1N2

The study conducted by Bravo-Vasquez et al., 2016 [30] to assess the exposure of backyard poultry and swine in the BPS of the El Yali wetland ecosystem to IAV found 42% of 31 BPS seropositive for IAV (with a 95% confidence interval ranging from 22% to 49%). During the sampling phase in 2014, involving 40 BPS, the seropositivity rate increased to 60% (with a 95% confidence interval ranging from 43% to 72%). All nine BPS exhibited seropositivity in both sampling periods. The RT-qPCR assay revealed that 27% of the BPS (with a 95% confidence interval ranging from 14% to 39%) tested positive for the IAV M gene. The cycle threshold (Ct) values of these positive samples ranged from 33.69 to 37.82. However, though below the RT-qPCR Ct cutoff of 38, these values were still relatively high for subtyping or viral isolation. In addition, eight farms (accounting for 73% of the RT-qPCR-positive farms) tested positive for IAV through the enzyme-linked immunosorbent assay (ELISA) test. Notably, one BPS showed simultaneous positivity for IAV in multiple species, including poultry, swine, and geese. On this farm, an SIV was successfully identified and subtyped as H1N2 [30].

### 3.3. IAV H1/H3/H4/H9/H10/H12

A more recent study by Bravo-Vasquez et al., 2020 [31] analyzed 329 samples from different regions of Central Chile and described the different risk factors associated with contracting the influenza virus in backyard animal production systems. The study also identified various subtypes of the avian IAV, including H1, H3, H4, H9, H10, and H12, using hemagglutination inhibition and microneutralization assays according to WHO guidelines [45]. Concerning seroprevalence at the BPS level, the study found rates of 34.7% (95% CI: 23.1–46.2%), 19.7% (95% CI: 9.9–30.6%), and 11.7% (95% CI: 7.2–16.4%) for the Metropolitan, Valparaiso, and LGB O’Higgins regions, respectively. Regarding prevalence at the BPS level, it was 4.2% (95% CI: 0.0–8.8%), 8.2% (95% CI: 0.8–14.0%), and 9.2% (95% CI: 4.8–13.1%) for the corresponding regions.

## 4. Avian Influenza Virus Subtypes in Wild Birds

### 4.1. AIVs H13N2, H5N9, and H13N9

During 2007–2009, there were reports of dead birds found along the coastlines of the northern (Arica and Atacama) and central regions (Valparaíso) of Chile [32]. The dead birds were sampled as part of the surveillance program conducted by the SAG to detect IAVs. Using RT-qPCR, four infected birds were identified, and strains of AIV subtypes A/seagull/Chile/SAG14259/2007(H13N2), A/wild bird/Chile/1805/2008(H5N9), and A/seagull/Chile/5775/2009(H13N9) were isolated. Sequence analysis of gene segments from all these Chilean strains revealed closer similarity to AIV strains identified in aquatic birds from North America’s western, central, and eastern regions than to other South American strains found along the Pacific and Atlantic coasts. This suggests that the distribution of Chilean AIV strains may be associated with migratory flyways based on geographical regions. Additionally, these findings also indicate that the distribution of strains was independent in both the Chilean and South American contexts. The observed homogeneity and similarity to North American lineages are also evident in the A/black-bellied whistling duck/Colombia/1/2011(H5N2) strain [32].

### 4.2. AIVs H9N2, H9N7, H4N2, H7N3, H7N6, H5N3, H3N6, H11N9, H4N6, H7N3, and H1N1

Between 2012 and 2015, a study by Jiménez-Bluhm et al., 2018 [33] tested 4036 fresh fecal samples of wild birds in Central and Northern Chile using RT-qPCR targeting the IAV M gene. In total, 115 samples tested positive for IAV; the prevalence of AIV varied depending on the season and site of collection. A significant increase in positivity was observed during the summer/fall seasons, compared to the winter/spring seasons (*p* = 0.007), indicating a seasonal variation in the presence of the virus. Additionally, it was also noted that three sites, which were heavily sampled, accounted for over half of the total sampling effort and yielded 73% of the positive samples. Among the identified bird species, yellow-billed pintails (*Anas georgica*) and yellow-billed teals (*Anas flavrostris*) from the Anseriformes order exhibited the highest prevalence of AIV infection and displayed the greatest AIV strain diversity. Various other species were identified as hosts, such as the Chiloé wigeon (*Anas sibilatrix*), mallards (*Anas platyrhynchos*), red-fronted coot (*Fulica rufifrons*), oystercatchers (*Haematopus*), gulls (*Larus*), black-necked stilts (*Himantopus mexicanus*), gray plovers (*Pluvialis squatarola*), and whimbrels (*Numenius phaeopus*). Full genomic sequences were obtained from 16 isolates and partial sequences from 20 positive swab samples. The identified AIV strains exhibited diverse combinations of HA and NA subtypes, including H1, H3, H4, H5, H6, H7, H8, H9, H11, H13, N1, N2, N3, N6, and N9. These strains were reassortants with unique gene combinations that resembled viruses isolated from both North and South America, unlike IAVs isolated from wild birds in other South American countries, where the genes were most like viruses isolated from wild birds in either North America or South America [33].

### 4.3. AIVs H9N2, H9N7, H3N8, H13N9, H13N8, H5N3, H13N2, H7N9

The Lluta River wetland in Northern Chile, a vital ecosystem and water source, serves as a crucial location for AIV surveillance due to its role in migratory bird routes. A study to determine IAV prevalence, subtype diversity, and ecological factors influencing prevalence was conducted from September 2015 to October 2020, with fecal samples collected for RT-qPCR. The AIV prevalence was 2.07%, varying monthly. Multiple HA and NA subtypes were detected, and ten viruses were isolated and genetically analyzed, among which were low pathogenic strains such as H5, H7, and H9. Migratory birds, particularly the *Laridae*, *Scolopacidae*, and *Charadriidae* families, were important reservoirs. Three viral isolates were found in migratory species, two in Franklin’s gulls (H13N9) and one in a Grey plover (H9N7). Further, various bird species serving as potential reservoirs, comprising both migratory and resident birds, were identified, with the Chilean flamingo (*Phoenicopterus chilensis*) being a recently acknowledged host. This study highlights the Lluta wetland’s significance in AIV surveillance, especially for viruses from the Northern Hemisphere [34].

## 5. Highly Pathogenic Avian Influenza (HPAI) H5N1

All the circulating HPAI H5N1 viruses originated from influenza A/goose/Guangdong/1/1996 that only infected birds [46]. Clade 2.3.4.4 viruses originated from clade 2.3.4 and have an increased capacity to reassort, giving rise to H5Nx viruses with novel NA pairings [46]. The evolution of this lineage gave rise to clade 2.3.4.4b viruses, where the H5 re-acquired an N1 NA pairing [46]. Clade 2.3.4.4b viruses currently pose a serious threat to public health and food security due to their zoonotic potential and poultry economic losses worldwide [47]. These viruses have been spreading widely among wild birds in Europe [48], Asia [49], and Africa [50]. Normally, sporadic outbreaks occur in the autumn and subside by the following spring. However, since 2022, outbreaks have continued to occur during the summer in Europe and North America [51,52]. Thus, in early 2022, the HPAI H5N1 clade 2.3.4.4b was detected in North America, most likely introduced by migratory birds from Europe [53], and, starting in late 2022, it spread throughout South America [54]. The first detection of the virus in Chile was on 7 December 2022, in a Pelecanus thagus specimen [35]. In the subsequent months, AIV H5N1 infections were documented in various avian species, marine mammals, and one human case [19,36,40].

### 5.1. AIV H5N1 in Wild Birds

**Figure 2:**
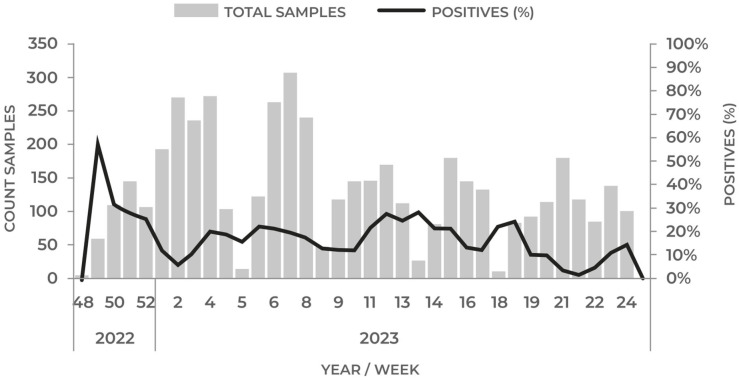
Positive prevalence of H5N1 cases in wild birds by epidemiological week. All samples were sequenced and categorized as the H5N1 subtype [36]. The timing of the sampling during the migration period could explain the higher prevalence of positive cases observed in the northern and central regions of the country (Figure 3).

In early December 2022, an increase in mortality was observed among wild birds, primarily pelicans, along the north coast of Chile. These birds were found either dead or moribund. From 1 December 2022 to 19 June 2023, a total of 4637 samples from wild birds originating from the entire territory of Chile were tested for HPAI by SAG [36]. Testing utilized the AGID test and RT-qPCR targeting the AIV M gene with the VetMAX-Gold AIV Detection Kit (Applied Biosystems™, Foster City, CA, USA). In total, 778 samples tested positive for AIV using RT-qPCR, resulting in a cumulative prevalence rate of 16.77%. The highest number of positive samples was recorded during week 49 of 2022, and the positivity rate remained consistent throughout the entire period, with an average prevalence of 17.0%. As shown in Figure 2, there was no apparent correlation between the prevalence and the number of samples analyzed. Notably, no positive AIV cases were detected in the 1080 domestic poultry samples that were tested in the same period.

Since 2015, extensive AIV surveillance in Chile focusing on wild birds and their potential interactions with domestic poultry and humans has been conducted by Jimenez-Bluhm et al., 2023 [37]. To enhance their monitoring efforts, they intensified surveillance in the Lluta River estuary by conducting biweekly sampling starting in August 2022. During August and September of 2022, the prevalence of AIV remained below 1%, as measured by M gene-specific qRT-PCR. However, there was a notable increase in November (2.6%) and December (11.7%), which coincided with the arrival of migratory birds from the Northern Hemisphere. Among the 69 AIV-positive environmental fecal samples collected in November and December (out of a total of 2023 samples), seven samples (10.1%) were identified as belonging to the H5 subtype. One sample was obtained in late November, while the remaining six were collected in December. Using Cytochrome Oxidase I speciation, the researchers determined that the H5-positive samples originated from various bird species, including the Peruvian pelican, Franklin’s gull, gray gull, elegant tern, and black skimmer. Three of the samples underwent full genome sequencing, resulting in the generation of two complete genomes from the gray gull and black skimmer. However, the Peruvian pelican sample only yielded a partial genome due to a low viral load. Phylogenetic analysis of the H5N1 sequences from Chile and the H5N1 viruses detected in pelicans and chickens in Peru during the same period indicated that the avian sequences from Chile formed a subcluster with Peruvian avian sequences sampled in late November 2022 [35], suggesting a common origin and adding to the massive mortality observed in over 3000 sea lions (*Otaria flavescens*) in Peru [18]. There were also fatal cases of sea lions in Chile [48]. Moreover, these Chilean–Peruvian subcluster sequences were located within a larger cluster that encompassed sequences from Ecuador, Mexico, and the USA [35]. These sequences were mostly sampled in late November 2022 in Ecuador and mid-October 2022 in Mexico and the USA, strengthening the notion that the Atlantic migratory route was the source of the introduction of the AIV H5N1 2.3.4.4b clade to the Americas.

**Figure 4:**
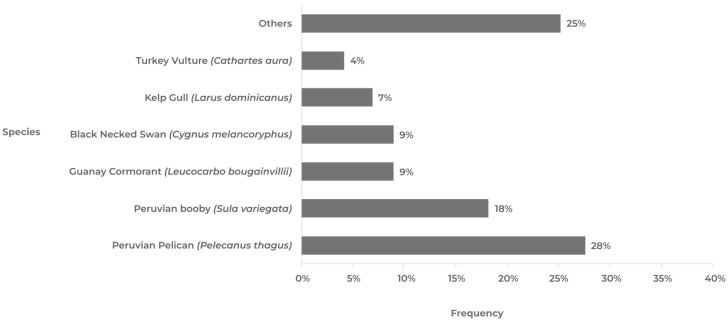
Frequency of avian influenza (H5N1) in wild birds, categorized by species. The prevalence of positive cases is represented, highlighting the species with the highest occurrence. Several of these species exhibit local or intercontinental migratory habits, which could explain the correlation of genotypes observed in the Northern Hemisphere. The data used for this analysis were derived from the official database published by the Chilean Agricultural and Livestock Service [36] between 5 December 2022 and 19 June 2023. The graph includes the frequency of positive cases for the Humboldt penguin (*Spheniscus humboldti*) and Magellanic penguin (*Spheniscus magellanicus*) under “Others”, which were examined as part of the official surveillance program conducted by the National Fisheries and Aquaculture Service [40].

The avian species in Chile with the highest positivity rates are linked to coastal waterways, and a significant number of them are migratory (Figure 4).

Among the wild birds with a higher prevalence of AIV positives, the species belonging to the orders Pelecaniformes (pelicans, herons, and ibises), Suliformes (cormorants and boobies), and Charadriiformes (gulls, terns, and sandpipers) stand out (Table 1). It has been observed that 75% of all AIV-positive avian cases are represented by a limited group of wild bird species. Humboldt’s pelican (*Pelecanus thagus*, Pelecaniformes) has shown the highest susceptibility, accounting for 28% of all positive cases. It is followed by the Peruvian booby (*Sula variegata*, Suliformes) and Guanay cormorant (*Leucocarbo bougainvillea*, Suliformes), which account for 18% and 9% of positive cases, respectively. Although these three species do not exhibit long-distance migratory behavior, their distribution ranges span a significant portion of the Pacific Coast (Chile, Peru, Ecuador, and Colombia), and they are frequently observed together in large flocks feeding near the coast [55].

On the other hand, specimens of the kelp gull (*Larus dominicanus*, Charadriiformes) and black-necked swan (*Cygnus melancoryphus*, Anseriformes) have a high frequency of positive cases, accounting for 9% and 7%, respectively. Among the long-distance migratory birds (USA–Chile), positive cases were detected in individuals of Franklin’s gull (*Leucophaeus pipixcan*) and sanderling (*Calidris alba*). Finally, although less frequently, positive cases were detected in groups of raptors such as the black vulture (*Coragyps atratus*), peregrine falcon (*Falco peregrinus*), and Chilean hawk (*Parabuteo unicinctus*) [36]. Concurrent with the detection and elevation of positive cases of avian flu H5N1 in wild birds, a substantial increase in both wild bird mortality and penguin strandings was noted [36,40] (Supplementary Table S2).

A spatiotemporal cluster analysis study of H5N1 cases in both wild and domestic birds in Chile was conducted by Azat et al., 2023 [38] using data covering the period from 9 December 2022 to 3 March 2023, reported by SAG to the World Animal Health Information System (WAHIS). The analysis identified 14 statistically significant clusters of H5N1 outbreaks, indicating the progression of the epidemic wave from the north to the south of Chile. These clusters were dispersed throughout the country, varying in size from two to 19 birds that tested positive for H5N1 in each cluster. Eight clusters had a radius of less than 1 km, while the remaining six clusters had radii ranging from 4 to 29 km. Notably, four clusters were located in the central zone of Chile, near densely populated areas, while one cluster (#12) was identified in the northern city of Tocopilla, where the first human case of H5N1 occurred at a later time. The study also revealed a robust linear correlation between distance and time since the first outbreak, suggesting the gradual, wave-like dissemination of H5N1. This relationship was further supported by the absence of spatial autocorrelation in the data, as indicated by the non-significant Global Moran’s I index. Additionally, the presence of H5N1 outbreaks in birds was found to be correlated with various ecological and human-related factors, including bird diversity, human activity, precipitation during the wettest month, the minimum temperature during the coldest month, and the mean diurnal temperature range. On the other hand, the presence of H5N1 was negatively correlated with the distance to the nearest urban center, precipitation seasonality, and isothermality. No significant associations were found between the presence of H5N1 and the annual mean temperature, temperature seasonality, precipitation variables, distance to the closest SAG office, human total population, or density. These findings highlight the complex interplay between ecological and human factors in the distribution and spread of HPAI H5N1 in Chile.

### 5.2. AIV H5N1 in Marine Mammals

**Figure 5:**
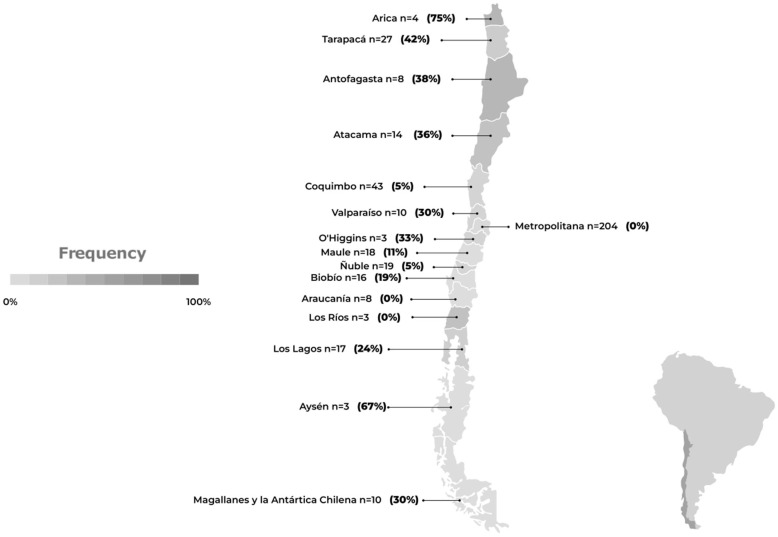
Avian influenza (H5N1) frequency in marine mammals in Chile. The number of samples and the frequency of positives are shown by region, based on data obtained from the official surveillance program of the National Fisheries and Aquaculture Service [40] between 15 February and 11 September 2023. The number of samples and frequency of positives for the Humboldt penguin (*Spheniscus humboldti*) and Magellanic penguin (*Spheniscus magellanicus*), analyzed in the official surveillance program of the National Fisheries and Aquaculture Service [40], are excluded from this graph, as they are considered in the wild bird graph.

Since the first case of HPAI H5N1 in wild birds in early December 2022, there has been constant occurrence of the virus affecting different species of marine mammals and birds across various regions of Chile (Table 2). Therefore, currently, there is a state of high alert due to reports of HPAI H5N1 in marine mammals, coinciding with the appearance of H5N1 in wild birds. The data presented in Figure 5 correspond to the total number of animals sampled and the percentages of animals that tested positive for AIV H5N1 in Chile, categorized by region. All data were collected by SERNAPESCA [40]. Based on these data, the regions of Tarapacá, Coquimbo, Maule, and Los Lagos had the highest number of cases sampled. However, it is worth noting that the virus had the highest prevalence, with a rate exceeding 20%, in the regions of Antofagasta, Atacama, and Biobío. This information provides an overview of the distribution of AIV H5N1 cases across different regions of Chile, highlighting both the quantity of sampled cases and the virus’s prevalence in each region.

**Figure 6:**
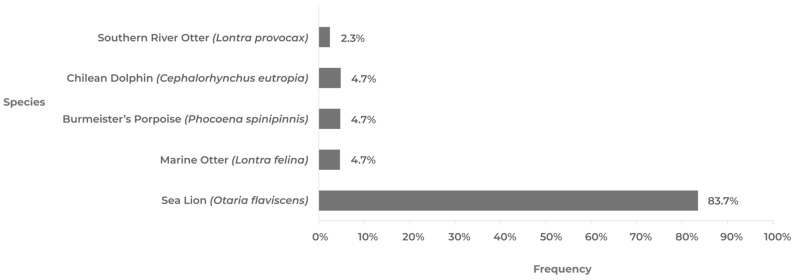
Frequency of avian influenza (H5N1) in marine mammals, categorized by species. The data used for this analysis were derived from the official surveillance program conducted by the National Fisheries and Aquaculture Service [40] between 15 February and 11 September 2023. The frequency of positives for the Humboldt penguin (*Spheniscus humboldti*) and Magellanic penguin (*Spheniscus magellanicus*), analyzed in the official surveillance program of the National Fisheries and Aquaculture Service [40], are excluded from this graph as they are considered in the wild bird graph.

On 15 February 2023, a sea lion (*Otaria flavescens*) in the Antofagasta region (Figure 6) became the first marine mammal in Chile to test positive for AIV H5N1. This is significant because sea lions are present in a large part of Chile’s continental territory and can travel over 200 km in search of food, making them highly susceptible to infection. To date, 32 out of over 144 sea lions sampled in Chile have been found positive for AIV H5N1. Another species of marine mammal is the Chungungo (*Lontra felina*), a carnivorous mammal found from Peru to Argentina. Recently, two cases of AIV H5N1 have been detected in Chungungos, in the northern regions of Chile. The Chilean dolphin (*Cephalorhynchus eutropia*) is an endemic species of marine mammal found in two locations in the Maule and Ñuble regions. Finally, the spiny porpoise (*Phocoena spinipinnis*) is a small cetacean found in the coastal waters of South America. Positive cases of this species have been reported from the Antofagasta and Atacama regions.

Since January 2023, a significant and sudden increase in strandings and mortality among South American sea lions (*Otaria flavescens*) has raised concern in Chile. This surge in mortality, peaking in June 2023 with 4545 stranded and deceased sea lions, coincides geographically with outbreaks of HPAIV H5N1 in wild birds. A comprehensive study investigated the cause, with 169 sea lions sampled for HPAIV H5N1 and an analysis of stranding trends from 2009 to 2023. Of the sampled sea lions, 20% tested positive for the influenza A virus. Clinical and pathological assessments of two necropsied sea lions revealed neurological and respiratory signs consistent with the observed mortality. The presence of the virus in the brain and lungs further supported the findings, suggesting a causal relationship between the mass sea lion mortality and HPAIV [39]. In Chile, as of August 2023, accumulated data indicate that 19,707 individuals of *Otaria flavescens* have been observed stranded. It is highly likely that this phenomenon is partially explained by HPAIV H5N1 infection (Supplementary Table S3) [40]. There is an emphasis on the need for more frequent clinical diagnostics to confirm HPAI H5N1 infection.

The transmission of IAVs among different animal species is a crucial factor in their evolution and ecology. The continued transmission of these viruses from bird species to marine mammal species may play an important role in the development of new viral strains [56]. The high number of seal cases in several countries in South America, and in the United States, Canada, the United Kingdom, and Russia, has led to the question of whether mammal-to-mammal transmission is involved or only avian-to-mammalian transmission [57]. The most sustained mammal-to-mammal transmission of the HPAI H5N1 clade 2.3.4.4b was the 2022 farmed mink outbreak in Spain [58], associated with several virus mutations [43]. Seal-to-seal transmission of HPAI H5N1 would not be surprising since it has occurred in Maine, US with low pathogenic influenza viruses, although available data do not support seal-to-seal transmission as a primary route of infection [57]. Furthermore, unlike other mammals known to contract H5N1, seals do not typically feed on birds [59], suggesting that if individual bird-to-seal spillover events were the primary transmission route, then the transmission occurred frequently and was associated with a low seal species barrier to HPAI H5N1 [57]. Additionally, following the interspecies transmission of IAVs, other factors, such as coinfection with other pathogens or predisposing environmental conditions, can enhance the virus’s pathogenicity for marine and terrestrial mammals.

The potential for marine mammals to host or carry zoonotic pathogens, such as HPAI H5N1, is a matter that requires further investigation. Moreover, the significant migration of marine mammals along the Chilean continental coast may increase the likelihood of direct contact with humans, thereby playing a key role in the spread of the H5N1 virus.

### 5.3. AIV H5N1 in Humans

On 29 March 2023, the Chilean Ministry of Health reported to the World Health Organization (WHO) the first confirmed case of human infection with the AIV H5 virus in the Antofagasta region [60]. This case represents the first documented occurrence of human infection with AIV H5 in Chile and the third in South America [19]. The patient from Chile was a 53-year-old man from the Antofagasta region. On 13 March 2023, he presented with symptoms of a cough and sore throat. There was no reported history of comorbidities or recent travel. As his health deteriorated, he was admitted to the Antofagasta Hospital on 21 March 2023. A nasopharyngeal swab was collected as part of regular monitoring for severe acute respiratory infections (SARIs). Subsequently, on 27 March, a bronchoalveolar sample was collected and tested positive for a non-sub-typeable IAV using RT-qPCR. The sample was sent to the Institute of Public Health (ISP), where it was confirmed to be positive for AIV H5 and belonging to the 2.3.4.4b clade [19]. Furthermore, its sequence was determined to be 99% identical to the H5N1 virus in samples found in wild birds in Chile. However, there is currently no evidence of human-to-human transmission of the circulating virus.

A recent study analyzed whole-genome H5N1 virus sequences from 77 birds, 8 marine mammals, and the human case [61]. Remarkably, two mutations in the PB2 segment (Q591K and D701N) associated with mammalian host adaptation were identified in Northern Chile in March 2023. D701N was described in four samples: one sanderling, two sea lions, and a human case. Two viruses with D701N also included the Q591K mutation, comprising the human case and a sea lion. Notably, these two substitutions showed a frequency of 0.2% for D701N and 0% for Q591K in H5N1 virus sequences collected globally, excluding Chile and Peru, since 01 January 2021. In Chile, although these four PB2 H5N1 virus sequences form a cluster, it remains unclear whether the D701N mutation was transmitted from sea lions or birds or whether these correspond to independent de novo emergence and there has been no transmission of D701N between hosts. The study by Pardo-Roa et al., 2023 [61] also revealed that the D701N substitution was present in 52.9–70.9% of sequence reads, signifying the coexistence of both genotypes in the host. Castillo et al., 2023 also confirmed the human case as subtyped in the clade 2.3.4.4b [62]. Additional zoonotic mutations have been described in other mammals [63]. The risk factors related to previously described detected AIVs encompass various elements, such as the extent of interaction between the infected humans and recipient animals, the compatibility of animal hosts for efficient virus infection, and the suitability of the animal host population for the ongoing virus presence. A major concern relates to the potential transmission of the virus to susceptible animal species, especially when these animals are in group housing situations and have contact networks that could potentially establish an alternative virus reservoir. This reservoir, in turn, could facilitate the reintroduction of the virus into the human population. In densely populated areas, virus exposure can lead to sustained transmission in susceptible animal species. To mitigate the risk of reverse zoonosis, it is essential to identify these risk factors and conduct serological surveillance in animal species susceptible to AIVs, particularly those housed in group settings. This proactive approach can help to mitigate the threat of virus transmission from animals to humans [64]. Some precedents describe how, during the artificial insemination process, turkey breeder hens may contract influenza virus from humans. Experimental infection has demonstrated that the virus tends to concentrate in the reproductive tract, with minimal spreading to other bodily tissues. These studies were conducted with a restricted number of hens, and the broader effects on the flock’s health, mortality, and reproductive capacity could not be conclusively ascertained [65].

## 6. Conclusions

In conclusion, this review summarizes historical and recent updates on the evolution of the different IAV subtypes in domestic poultry, wild birds, and mammals including humans, in Chile, since the first detection of IAV in commercial poultry in Chile in 2002. Over the last two decades, disease outbreaks in domestic and wild bird species have been caused by different IAVs genetically related to IAVs in North America. The HPAI H5N1 viruses first detected in North America in early 2022 after their introduction from Europe were subsequently detected in South America, including Chile, in commercial poultry, backyard poultry, wild birds, and mammals, including one human case. The distribution and spread of AIV H5N1 in Chile indicated a complex interplay between ecological and human factors in that it was negatively correlated with the distance to the closest urban center and precipitation and temperature seasonality, suggesting a potential to cross over to Antarctica/subantarctic islands such as Malvinas. It is evident that HPAI H5N1 in Chile was introduced via the Atlantic flyway, which has been shown as an important pathway leading to the AIV diversity in Chile, as opposed to local transmission from other countries in South America. The presence of these viruses in Chile underscores the need for increased biosecurity on poultry farms and continuous genomic surveillance approaches to understand and control AIVs in both wild and domestic bird populations in Chile, which appear to form a distinct cluster to the South American AIVs. Building on the findings presented in this review of HPAI H5N1 and IAV evolution in Chile, it is imperative to outline four key strategies for future endeavors in avian influenza management.

- Proactive surveillance of migratory birds: As we anticipate upcoming migratory seasons, it is essential to implement a proactive strategy encompassing enhanced detection and genomic surveillance, to ensure the effective control and monitoring of wild birds. Continued monitoring of critical locations along migratory routes, such as the Lluta River wetland in Northern Chile, remains crucial to early detection and intervention.
- Comprehensive stranding control: Addressing avian influenza also necessitates a holistic approach including not only the control of stranding events but also the systematic detection and subtyping of AIVs in the affected marine mammals and bird populations. This multifaceted approach can provide valuable insights into the diversity and dynamics of the virus.
- Expanded backyard poultry surveillance: Given the evident role of wild birds and marine mammals in virus dissemination, it is imperative to expand surveillance efforts for avian influenza in backyard poultry across various regions of the country. This broader approach should encompass areas influenced by both migratory birds and marine mammals, recognizing their potential as secondary vectors.
- Coordinated genomic surveillance for one health: Effective risk mitigation necessitates a coordinated One Health approach that integrates the genomic surveillance of avian influenza, addressing both human and animal health concerns. This approach should be underpinned by robust communication channels facilitating collaboration among diverse stakeholders from the public and private sectors involved in managing the disease across different compartments.

Incorporating these strategic measures into future avian influenza management efforts will be essential for enhancing preparedness, early detection, and control of the virus in Chile. By addressing both the ecological and genomic aspects of avian influenza, a more comprehensive and effective approach can be achieved. Furthermore, a previous systematic review of studies on viral and bacterial pathogens in wild birds in Chile [66] noted that *Salmonella* spp. and AIV have been the most studied pathogens, but it also highlighted a lack of research coverage in various regions of the country. This underscores the importance of implementing these proposed strategies to comprehensively address avian influenza in Chile and enhance our understanding and control of this pathogen.

## Figures and Tables

**Figure 3:**
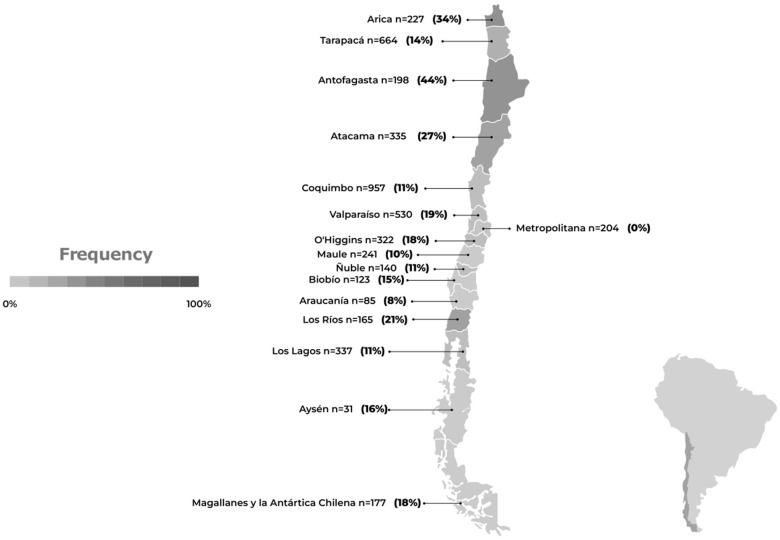
Avian influenza (H5N1) frequency in wild birds in Chile. The number of samples and the frequency of positives are shown by region, based on data obtained from the official surveillance program of the Chilean Agricultural and Livestock Service [36] between 5 December 2022 and 19 June 2023. The number of samples and frequency of positives for the Humboldt penguin (*Spheniscus humboldti*) and Magellanic penguin (*Spheniscus magellanicus*), analyzed in the official surveillance program of the National Fisheries and Aquaculture Service [40], are also included.

**Table 1:** Number of wild birds positive for influenza A virus by species and geographical region.

Region	Peruvian Pelican (*Pelecanus thagus*)	Peruvian Booby (*Sula variegata*)	Guanay Cormorant (*Leucocarbo bougainvillii*)	Kelp Gull (*Larus dominicanus*)	Black-Necked Swan (*Cygnus melancoryphus*)	Turkey Vulture (*Cathartes aura*)	Others	Total
Arica	7	18	12	4	0	2	34	77
Tarapacá	8	19	29	4	0	8	23	91
Antofagasta	31	23	2	0	0	13	16	85
Atacama	41	24	7	6	0	2	10	90
Coquimbo	21	33	12	14	0	2	22	104
Valparaíso	62	13	3	3	1	3	16	101
O’Higgins	21	8	5	6	4	3	11	58
Maule	9	3	0	3	0	0	9	24
Ñuble	10	0	0	6	0	0	0	16
Bío Bío	5	1	0	6	0	0	6	18
Araucanía	0	0	0	0	3	0	4	7
Los Ríos	0	0	0	0	30	0	5	35
Los Lagos	1	0	0	1	22	0	12	36
Aysen	0	0	0	0	0	0	5	5
Magallanes	0	0	0	1	10	0	20	31
Total	216	142	70	54	70	33	193	778

The number of samples and the frequency of positives are shown by region based on data obtained from the official surveillance program of the Chilean Agricultural and Livestock Service [36] between 5 December 2022 and 19 June 2023. The number of samples and frequency of positives for Humboldt penguin (*Spheniscus humboldti*) and Magellanic penguin (*Spheniscus magellanicus*) analyzed in the official surveillance program of the National Fisheries and Aquaculture Service [40] are also included. Others include 44 avian species.

**Table 2:** Number of marine mammals positive for influenza A virus by species and geographical region.

Region	Marine Otter (*Lontra felina*)	Chilean Dolphin (*Cephalorhynchus eutropia*)	Southern River Otter (*Lontra provocax*)	Sea Lion (*Otaria flavescens*)	Burmeister’s Porpoise (*Phocoena spinipinnis*)	Total
Arica	1	0	0	2	0	3
Tarapacá	1	0	0	10	0	11
Antofagasta	0	0	0	2	1	3
Atacama	0	0	0	4	1	5
Coquimbo	0	0	0	2	0	2
Valparaíso	0	0	0	3	0	3
O’Higgins	0	0	0	1	0	1
Maule	0	1	0	1	0	2
Ñuble	0	1	0	0	0	1
Bío Bío	0	0	0	3	0	3
Araucanía	0	0	0	0	0	0
Los Ríos	0	0	0	0	0	0
Los Lagos	0	0	0	4	0	4
Aysén	0	0	0	2	0	2
Magallanes	0	0	1	2	0	3
Total	2	2	1	36	2	43

The number of samples and the frequency of positives are shown by region based on data obtained from the official surveillance program of the National Fisheries and Aquaculture Service [40] between 15 February and 11 September 2023.

## Data Availability

The datasets analyzed during the current study are available from SAG (https://www.sag.gob.cl/ia), SERNAPESCA (http://www.sernapesca.cl/influenza-aviar), NCBI (https://www.ncbi.nlm.nih.gov/), and from the corresponding authors on reasonable request. All three datasets were accessed in September 2023.

## Supplementary Materials

The following supporting information can be downloaded at: https://www.mdpi.com/article/10.3390/pathogens12101252/s1; Table S1: List of influenza A virus isolates from avian hosts in Chile accumulated up to September 2023. Table S2: Level of wild bird mortality by species and geographical region accumulated up to May 2023 and penguin strandings accumulated up to September 2023. Table S3: The number of marine mammal strandings by species and geographical region accumulated up to September 2023.
